# Repeatability and reproducibility of cardiac manganese-enhanced magnetic resonance imaging

**DOI:** 10.1038/s41598-023-29591-z

**Published:** 2023-02-27

**Authors:** T. Singh, S. Joshi, M. N. Meah, N. B. Spath, G. Papanastasiou, L. E. Kershaw, A. H. Baker, M. R. Dweck, D. E. Newby, S. I. Semple

**Affiliations:** 1 BHF/University Centre for Cardiovascular Science, University of Edinburgh, Edinburgh, UK; 2 Edinburgh Heart Centre, Royal Infirmary of Edinburgh, Edinburgh, UK; 3 Edinburgh Imaging, University of Edinburgh, Edinburgh, UK

**Keywords:** Cardiology, Acute coronary syndromes, Cardiomyopathies, Heart failure

## Abstract

Manganese-enhanced magnetic resonance imaging can provide a surrogate measure of myocardial calcium handling. Its repeatability and reproducibility are currently unknown. Sixty-eight participants: 20 healthy volunteers, 20 with acute myocardial infarction, 18 with hypertrophic and 10 with non-ischemic dilated cardiomyopathy underwent manganese-enhanced magnetic resonance imaging. Ten healthy volunteers were re-scanned at 3 months. Native T1 values and myocardial manganese uptake were assessed for intra and inter-observer repeatability. Scan-rescan reproducibility was assessed in ten healthy volunteers. Intra-observer and inter-observer correlation was excellent in healthy volunteers for mean native T1 mapping [Lin’s correlation coefficient (LCC) 0.97 and 0.97 respectively] and myocardial manganese uptake (LCC: 0.99 and 0.96 respectively). Scan-rescan correlation for native T1 and myocardial manganese uptake was also excellent. Similarly, intra-observer correlations for native T1 and myocardial manganese uptake in patients with acute myocardial infarction (LCC: 0.97 and 0.97 respectively), hypertrophic (LCC: 0.98 and 0.97 respectively) and dilated cardiomyopathy (LCC: 0.99 and 0.95 respectively) were excellent. Limits of agreement were broader in patients with dilated cardiomyopathy. Manganese-enhanced magnetic resonance imaging has high repeatability and reproducibility in healthy myocardium and high repeatability in diseased myocardium. However, further study is needed to establish robustness in pathologies with diffuse myocardial fibrosis.

## Introduction

Cardiac magnetic resonance imaging has a major role in the diagnosis, evaluation of myocardial function and tissue characterization of a range of cardiovascular diseases^1–3^. Conventional cardiac magnetic resonance with gadolinium enhancement allows for quantification of myocardial fibrosis which has utility in the assessment of viability in ischemic cardiomyopathy. Furthermore, it allows for prognostication in a variety of cardiac conditions including myocarditis, dilated cardiomyopathy, hypertrophic cardiomyopathy, amyloidosis^4–6^, arrhythmogenic right ventricular dysplasia^7^ and Fabry’s disease^8^. However, gadolinium-based contrast media only allow for the assessment of the extracellular space.

Magnetic resonance imaging using manganese-based contrast media has the ability to provide intracellular contrast of viable myocardium. Manganese was the first clinical magnetic resonance imaging contrast medium to be used in vivo, resulting in shortening of T1 relaxation in the heart, liver, kidneys and pancreas^9^. In brief, after intravenous administration in humans, manganese dipyridoxyl diphosphate undergoes dephosphorylation and transmetallation with zinc to release manganese ions into the plasma^10,11^. Being, a calcium analogue, manganese is actively taken up by voltage-gated calcium channels in viable myocardium whereas abnormal myocardium has reduced or no uptake. Myocardial manganese uptake can be calculated using Patlak kinetic modelling^12–14^, thereby providing a measure for myocardial calcium handling^15,16^. Indeed, manganese-enhanced T1 mapping can detect dysfunctional myocardial calcium handling in patients with cardiomyopathies and can distinguish between normal and pathological myocardium^16–18^

Repeatability of the assessment of manganese-enhanced magnetic resonance imaging and its scan-rescan reproducibility have not been established and is a necessary step for its future clinical application. The aims of this study were to establish the intraobserver and interobserver repeatability of manganese-enhanced T1 mapping and kinetic modelling of myocardial manganese uptake as well as provide early data on overall scan-rescan reproducibility.

## Methods

### Ethics approval and consent to participate

The study was conducted in accordance with the Declaration of Helsinki, with the favourable ethical opinion of the South-east Scotland Research Ethics Committee 2 (MEMRI-17/SS/0055, MEMORY-20/SS/0001) and with the written informed consent of all participants.

### Study population

Adult (≥ 18 years of age) healthy volunteers (n = 20) were recruited as part of the MEMORY study [NCT04623788]. Patients with acute myocardial infarction (n = 20), hypertrophic cardiomyopathy (n = 18) or non-ischemic dilated cardiomyopathy (n = 10) were recruited from the Edinburgh Heart Centre as part of the MEMRI study [NCT03607669]. Patients with acute myocardial infarction were required to have a ST-segment elevation myocardial infarction according to the universal definition of myocardial infarction^19^ and angiographically proven coronary artery disease. Patients were required to be clinically stable with reduced left ventricular ejection fraction (≤ 50% by echocardiography) secondary to one or more acute ischaemic events.

The diagnosis of hypertrophic cardiomyopathy and dilated cardiomyopathy were based on echocardiography or magnetic resonance imaging according to European Society of Cardiology guidelines^20,21^. Hypertrophic cardiomyopathy was defined as left ventricular hypertrophy (left ventricular wall thickness ≥ 15 mm in any segment) in the absence of hemodynamic stresses^21^. Non-ischemic dilated cardiomyopathy was defined by the presence of impaired left ventricular systolic function (ejection fraction ≤ 50% within 12 months) and left ventricular dilatation (left ventricular end-diastolic volume > 105 mL/m^2^ for men and > 96 mL/m^2^ for women, adjusted for age and body-surface area), in the absence of abnormal loading conditions (hypertension and valvular disease) and coronary artery disease^20^.

### Magnetic resonance imaging

All participants were scanned on a Siemens MAGNETOM Skyrafit 3 T scanner (Siemens Healthineers, Erlangen, Germany) with a 30-channel body matrix coil. Electrographic gated breath-hold steady-state free procession long-axis cine images in two, three and four chamber views were acquired. Short axis cine images covering the entire left ventricle were taken at 8-mm slice thickness, 2-mm gap, field of view 300 × 400 mm, matrix 208 × 256, repetition time 2.9 ms, echo time 1.2 ms, flip angle 64–790, temporal resolution < 50 ms, with 30 phases per cardiac cycle, in-plane image resolution 1.1 × 1.5 mm to 1.3 × 1.7 mm.

All participants underwent gadolinium enhanced magnetic resonance imaging, followed by manganese-enhanced imaging. This was performed at least 48 h apart. Additionally, healthy volunteers underwent repeat manganese-enhanced magnetic resonance imaging, 3 months following baseline imaging.

### T1 mapping

For healthy volunteers, T1 mapping was acquired with a modified Look-Locker inversion recovery sequence^22,23^ and in patients, T1 mapping was acquired with shortened modified Look-Locker inversion recovery [(WIP #1048 Siemens Healthineers), with a 5(3)3] sampling pattern and the following typical parameters: slice thickness 8.0 mm with 1.6-mm gap, field of view = 360 × 280 mm, repetition time 388.8 ms; echo time 1.07 ms, matrix 256 × 115. To minimize image artefacts, acquisition was performed with the region of interest (the heart) at isocentre, a small shim volume applied around the myocardium, and a large field of view (400 mm). All T1 mapping was acquired with non-rigid motion correction. The same parameters were used for scan-rescan reproducibility with the chosen slice being matched visually by the supervising cardiologist using appropriate anatomical landmarks to ensure the same location was being analysed.

### Late gadolinium enhancement

Late gadolinium enhancement images were acquired following intravenous gadobutrol (0.1 mmol/kg; Gadovist, Bayer, Germany) using a single breath hold per slice with a short-axis stack, and long-axis orientations. A full short-axis T1 stack was acquired prior to and 10 min after contrast administration as described previously^16,17^.

### Manganese infusion

Manganese-enhanced magnetic resonance imaging was conducted with intravenous infusion of manganese dipyridoxyl diphosphate [5 μmol/kg (0.1 mL/kg) at 1 mL/min; Exova SL Pharma, Wilmington, Delaware, USA] and has been described previously^16,17^. Following a full short-axis native T1 stack, a single mid-ventricular short-axis slice was identified and performed at this location every 2.5 min for 30 min after starting manganese infusion, at which point a full short-axis T1 stack was repeated (Supplemental Fig. S1).

**Figure 1:**
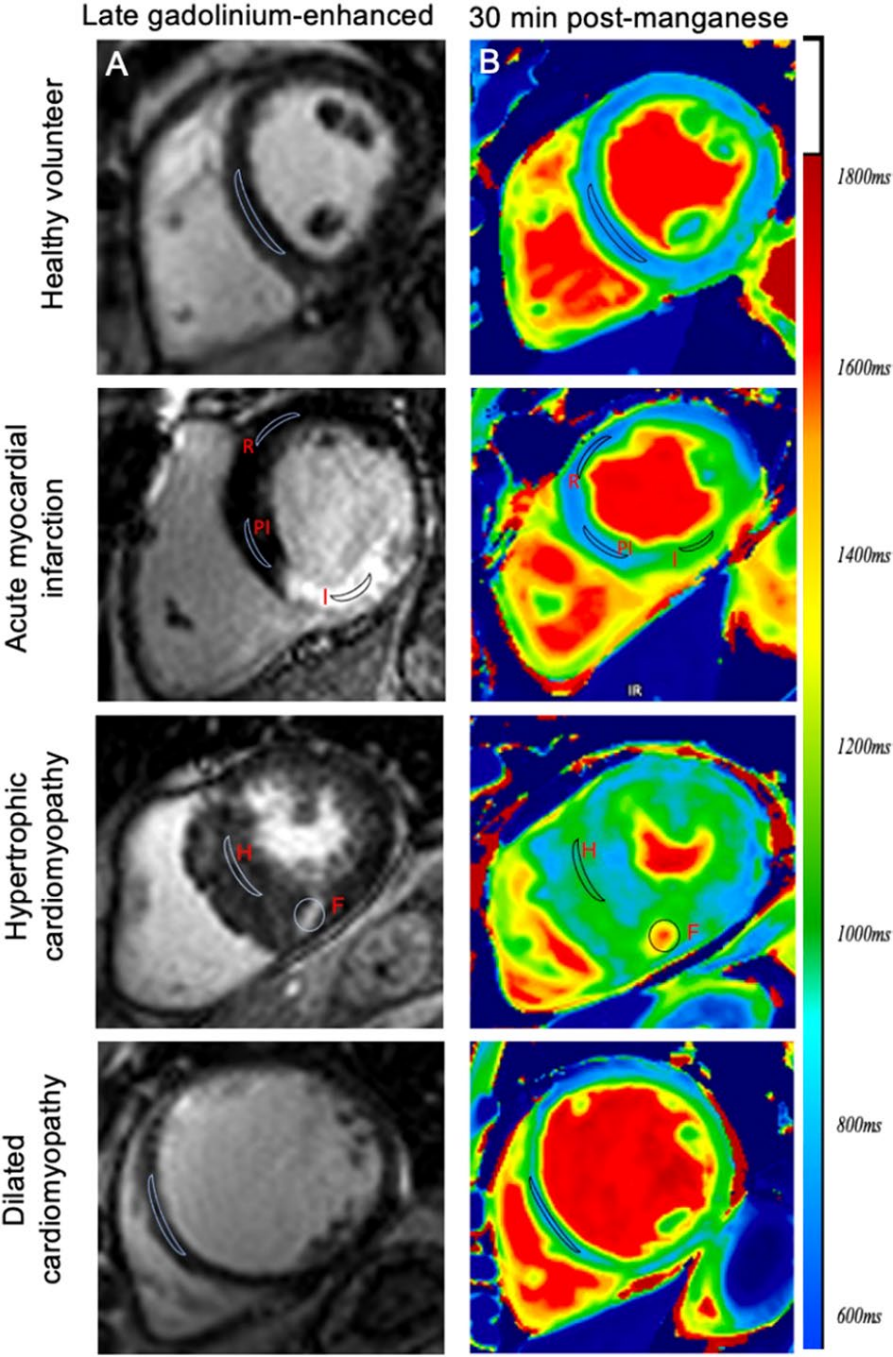
Regions of interest in managanese-enhanced magnetic resonance imaging. Late-gadolinium enhanced (**A**) and 30 min post-manganese T1 maps (**B**) in healthy volunteers, patients with acute myocardial infarction, hypertrophic and dilated cardiomyopathy. Demonstrating regions of interest in healthy volunteers (septum) and patients with acute myocardial infarction (infarct, I, peri-infarct, PI, and remote, R), hypertrophic cardiomyopathy (hypertrophied, non-fibrotic, H and fibrotic, F) and dilated cardiomyopathy (septum).

A single mid-ventricular slice was chosen for healthy volunteers. For patients, the short-axis slice was identified by the supervising cardiologist, guided by late gadolinium enhancement imaging, native T1 maps and cine images to represent abnormal myocardium (Fig. 1). For patients with acute myocardial infarction, infarct area was assessed by late-gadolinium images and to reduce variability, automated reference regions of interest were generated in the infarct region.

For patients with hypertrophic cardiomyopathy, regions of maximal hypertrophy and fibrosis were selected and for dilated cardiomyopathy, a mid-ventricular short-axis slice was selected. The chosen slice was matched visually by the same supervising cardiologist for repeat scanning.

### Image analysis

All analyses of T1 maps, late gadolinium enhancement and cine-derived volumetric and functional sequences were performed using Circle CVI (Circle Cardiovascular Imaging, CVI42 v5.3.6, Calgary Canada) as described previously^16,17^. Image analysis was performed by two MRI trained observers who were blinded to participant details.

All images were assessed for artefacts caused by susceptibility effects and cardiac or respiratory motion. The presence of artefacts led to the exclusion of all affected myocardial segments. Endocardial and epicardial borders were manually defined on all conventional short-axis images for volumetric and wall motion measurements and were then copied to corresponding T1 map images for analysis with minimal manual adjustments. The left ventricular basal short axis slice was identified as the image containing at least 50% of circumferential myocardium at end diastole. Papillary muscles were included in the mass and excluded from volumetric analysis.

Segmental variation in native and post-manganese T1 and myocardial manganese uptake (Ki) was only assessed in healthy volunteers. T1 values were derived from segments 7–12 (mid-ventricular slice) of a standard 16-segment model, as well as septal (region of interest in mid-septal wall) and global (average of all 6 segments from mid-ventricular slice) values. After contouring, an additional epicardial and endocardial offset of 20% was applied automatically to minimize partial volume effect for all T1 map analyses.

For patients with myocardial infarction, a reference region of interest was manually generated in the remote myocardium, with minimal manual adjustment based on the opposing wall from the late-gadolinium enhancement-defined infarct and wall motion by cine sequences where necessary. Given the lack of established consensus on quantification, a threshold of 2 × SD above remote myocardium was used for area at risk^24^. Peri-infarct tissue was defined as late-gadolinium enhancement negative but with elevated T1 in the area at risk (> 2 × SD) in the infarct related artery territory. For patients with hypertrophic cardiomyopathy, regions of interest were drawn in areas with hypertrophy and fibrosis (guided by late gadolinium enhancement). In patients with dilated cardiomyopathy, regions were drawn in the mid-septal wall, due to lack of late-gadolinium enhancement (Fig. 1). For serial T1 imaging post manganese, manually drawn regions of interest from the pre-contrast image were transferred to all subsequent post-contrast images to ensure consistency.

### Intra and inter-observer reproducibility

To test for intra-observer variability, scans were analysed in a random order, twice by the same operator, 6 months apart to reduce the risk of recall bias. To test for inter-observer variability, ten random datasets from each patient cohort and healthy volunteer data set were analysed by a second observer.

### Scan–rescan repeatability

Scan-rescan analysis was performed on a subset of healthy volunteers (n = 10) who underwent repeat manganese-enhanced cardiac magnetic resonance imaging 3 months after baseline scanning.

### Kinetic modelling

To derive quantitative estimates and to assess differential manganese uptake, kinetic model analysis was performed, as described previously^16,17,25^. Kinetic modelling was based on a Patlak two-compartment model formulation^12,14^. In brief, the model consists of (i) a reversible compartment (*v*_e_), comparable to intravascular and interstitial space and (ii) an irreversible compartment (*v*_i_) comparable to the intracellular space, in which irreversible accumulation of the contrast agent is anticipated during the imaging period (30 min). The arterial concentration (derived from blood pool T1) represents contrast agent delivery into myocardial tissue and constitutes the arterial input function.

Skjöld et al.previously derived a Patlak model formulation for cardiac manganese-enhanced magnetic resonance imaging^14^, demonstrating that an apparent unidirectional influx constant (*Ki*) for the transfer of manganese from plasma to irreversible compartments *v*_i_, can be measured, using Eq. (1):

1 $$\frac{{C}_{t}\left(t\right)}{{C}_{a}\left(t\right)}=Ki\frac{{\int }_{0}^{t}{C}_{a}\left(\tau \right)d\tau }{{C}_{a}\left(t\right)}+{v}_{e}$$

where *C*_t_ and *C*_a_ are the manganese concentration in myocardial tissue and blood pool (arterial input function) respectively. This formulation is equivalent to the Patlak model ^12^ and describes that if a contrast medium is irreversibly trapped in the tissue within the imaging period, the instantaneous tissue concentration divided by the instantaneous arterial concentration plotted against the integrated arterial concentration divided by the instantaneous arterial concentration, will result in linearization of the data. The gradient of this line represents the apparent unidirectional influx constant *Ki*, which equals:

2 $${K}_{i}=\frac{{k}_{1} \cdot {k}_{3}}{{k}_{2}+ {k}_{3}}$$

where *k*1, *k*2, and *k*3 are the individual rate constants of the compartmental model presented. A visual representation of the influx constant Ki is given in Supplementary Fig. S2.

### Statistical analysis

Data are expressed as mean ± standard deviation or mean (95% confidence interval) for continuous variables or median [interquartile range] where not normally distributed. Categorical variables are presented as number (percentage). Data were analysed using paired or unpaired Student’s *t*-tests, mixed effects model, linear regression analysis and Lin’s concordance correlation coefficients. Group variance was examined with the Brown-Forsythe test. Coefficient of variation (%) was defined as the average of means divided by the standard deviation of mean difference. Repeatability and reproducibility were determined using Bland–Altman analysis and bias (mean difference) is presented alongside 95% limits of agreement. Statistical analysis was performed using GraphPad Prism (Version 8.0, GraphPad Software, San Diego, California, USA). Statistical significance was taken as a two-sided P*-*value < 0.05.

## Results

### Manganese infusion

Seventy-eight infusions of manganese dipyridoxyl diphosphate were completed during the course of the study (mean duration, 10 min). There were no changes in the electrocardiogram, heart rate or blood pressure following manganese dipyridoxyl diphosphate administration (P > 0.1 for all). Two healthy volunteers and one patient with dilated cardiomyopathy experienced mild transient nausea for < 10 s after commencing manganese dipyridoxyl diphosphate infusion, spontaneously resolving without intervention. Otherwise, administration of manganese dipyridoxyl diphosphate was well tolerated with no adverse reactions reported during or immediately after administration or after seven days of follow up.

### Healthy volunteers

#### Intra-observer repeatability

In twenty healthy volunteers, a total of 120 segments were analysed. Six segments, predominantly the infero-lateral wall (segment 11), were excluded due to artefact. The mean septal native T1 was 1218 ± 24 ms (Supplementary Fig. S3). There was regional difference in myocardial native T1, with septal T1 appearing to be higher than global T1 (1218 ± 24 versus 1215 ± 22 ms, P = 0.02, Supplementary Fig. S3). Furthermore, myocardial manganese demonstrated similar segmental variation across the myocardium (P = 0.04, Supplementary Fig. S3).

There were no differences between mean myocardial septal native and post-manganese T1 on repeat analysis by the same observer (p = 0.75 and 0.63 respectively), with excellent correlation (Lin’s concordance correlation: 0.97 and 0.95 respectively, Table 1). Bland–Altman plots demonstrate excellent intra-observer agreement; however, limits of agreement (LoA) were wider for post-manganese T1 compared with native T1 (880 ± 24 ms, bias: + 3.0 ms, LoA: −9.1 to 14.8 versus 1218 ± 24 ms, bias + 2.2 ms, LoA: −12.5 to 8.0, respectively, Table 1).

**Table 1:** Intra-observer, inter-observer repeatability and scan-rescan reproducibility for healthy.

Intra-observer (*n* = 20)	First measurement	Second measurement	Mean difference	P value	LCC (95% CI)	Bland–Altman 95% LoA (95% CI)	CoV (%)
Native T1 (ms)	1218 ± 24 [1189–1270]	1220 ± 22 [1187–1273]	+ 2.2	0.75	0.97 (0.92–0.99)	−12.5 (16.8, −8.2) to 8.0 (3.7, 12.3)	1.3
30-min post manganese T1 (ms)	880 ± 24 [823–922]	883 ± 17 [851–918]	+ 3.0	0.63	0.95 (0.78–0.97)	−9.1 (9–14, −3.9) to 14.8 (9.6, 19.9)	3.3
Manganese uptake (Ki, mL/100 g of tissue/min)	8.4 ± 0.7 [7.2–10.2]	8.3 ± 0.7 [8.0–8.7]	−0.01	0.91	0.99 (0.95–0.99)	− 0.6 (−0.8, −0.3) to 0.5 (0.31, 0.79)	7.1

Inter-observer (*n* = 20)	First observer	Second observer					
Native T1 (ms)	1218 ± 24 [1189–1270]	1221 ± 27 [1183–1279]	+ 2.9	0.96	0.98 (0.91–0.99)	− 9.1 (−14, −3.9) to 14.8 (9.6, 19.9)	1.8
30-min post manganese T1 (ms)	880 ± 24 [838–930]	885 ± 28 [809–933]	+ 4.9	0.80	0.91 (078–0.96)	− 19.7 (−23.4, −9.4) to 29.3 (34.5, 15.3)	3.9
Manganese uptake (Ki, mL/100 g of tissue/min)	8.4 ± 0.7 [7.2–10.2]	8.2 ± 0.7 [7.1–10.0]	−0.04	0.96	0.96 (0.91–0.99)	− 0.7 (−0.9, −0.3) to 0.6 (0.4, 0.90)	8.9

Scan–rescan (*n* = 10)	Baseline scan	Repeat scan					
Native T1 (ms)	1230 ± 23 [1213–1247]	1226 ± 15 [1213–1239]	−4.0	0.60	0.94 (0.76–0.98)	−12.6 (−25.1, −0.07) to 22.6 (10.1, 35.3)	1.4
30-min post manganese T1 (ms)	899 ± 28 [871–927]	894 ± 27 [867–921]	−4.5	0.71	0.92 (0.64–0.95)	−26.8 (−43.2, −12.6) to 15.7 (0.15, 31.3)	2.6
Manganese uptake (Ki, mL/100 g of tissue/min)	8.4 ± 0.7 [8.0–8.6]	8.5 ± 0.7 [7.9–9.1]	+ 0.04	0.89	0.97 (0.91–0.99)	− 0.3 (−0.6, −0.1) to 0.3 (−1.7, 1.5)	8.6

Mean ± standard deviation [95% confidence interval].

*LCC* Lin’s concordance correlation, *LoA* limits of agreement, *CoV* coefficient of variation.

**Figure 2:**
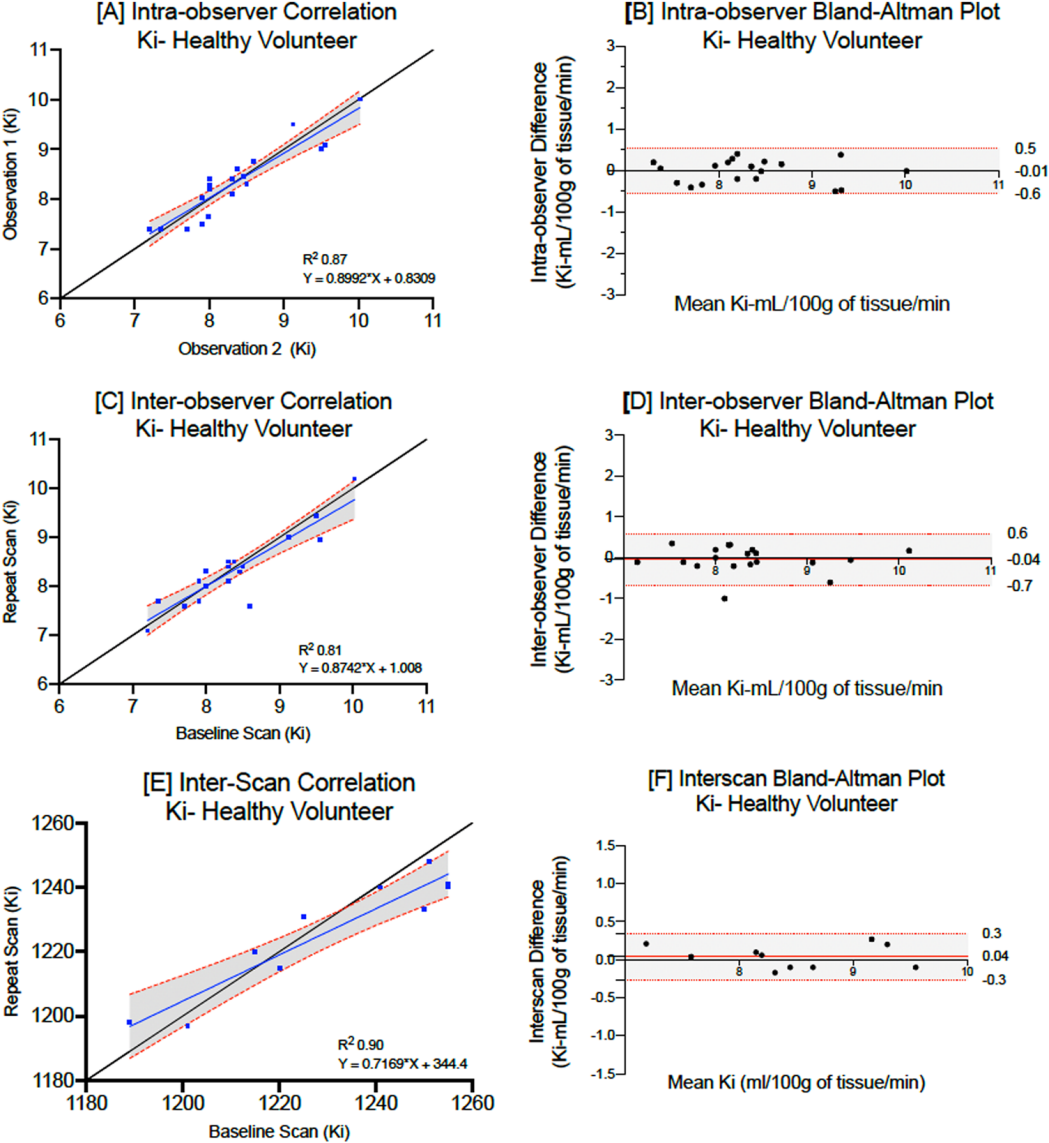
Intra, inter- observer repeatability and scan–rescan reproducibility for myocardial manganese uptake in healthy volunteers. Linear regression analysis and Bland–Altman plots demonstrating intra-observer repeatability (**A,B**), inter-observer repeatability (**C,D**) and scan–rescan reproducibility (**E,F**) in healthy volunteers.

Mean myocardial manganese (septal) uptake in healthy volunteers was 8.4 ± 0.7 mL/100 g of tissue/min and similarly had excellent correlation on repeated measurement (Lin’s correlation coefficient: 0.99, Table 1). Coefficient of variation was higher for manganese uptake compared to native and post-manganese T1 (7.1, 1.3 and 3.3% respectively). Despite this, Bland–Altman plots highlighting limits of agreement (Fig. 2) demonstrate excellent intra-observer agreement.

#### Inter-observer repeatability

There were no differences in myocardial native T1 (1218 ± 24 versus 1221 ± 27 ms, bias: + 2.9 ms, p = 0.96) and estimates of mean myocardial manganese uptake (8.4 ± 0.7 versus 8.2 ± 0.7 mL/100 g of tissue/min, bias: − 0.04, p = 0.96) in healthy volunteers, with all showing excellent correlations (Table 1). Bland–Altman plots highlight excellent inter-observer agreements for native T1, post-manganese T1 and myocardial manganese uptake (Fig. 2).

#### Scan–rescan reproducibility

Ten healthy volunteers underwent repeat manganese-enhanced imaging 88 [range: 62–124] days following baseline imaging. There were no differences between repeated scans for mean native T1 (1230 ± 23 versus 1226 ± 15, P = 0.60) and myocardial manganese uptake (8.4 ± 0.7 versus 8.5 ± 0.7, P = 0.89, Table 1). There was excellent correlation between the paired scans for mean native T1 and myocardial manganese uptake (Lin’s correlation coefficient: 0.94 and 0.97 respectively, Table1). Similarly, Bland–Altman plots demonstrate narrow limits of agreement between the two paired scans (Fig. 2, Table 1).

### Patient cohorts

#### Intra-observer repeatability

**Figure 3:**
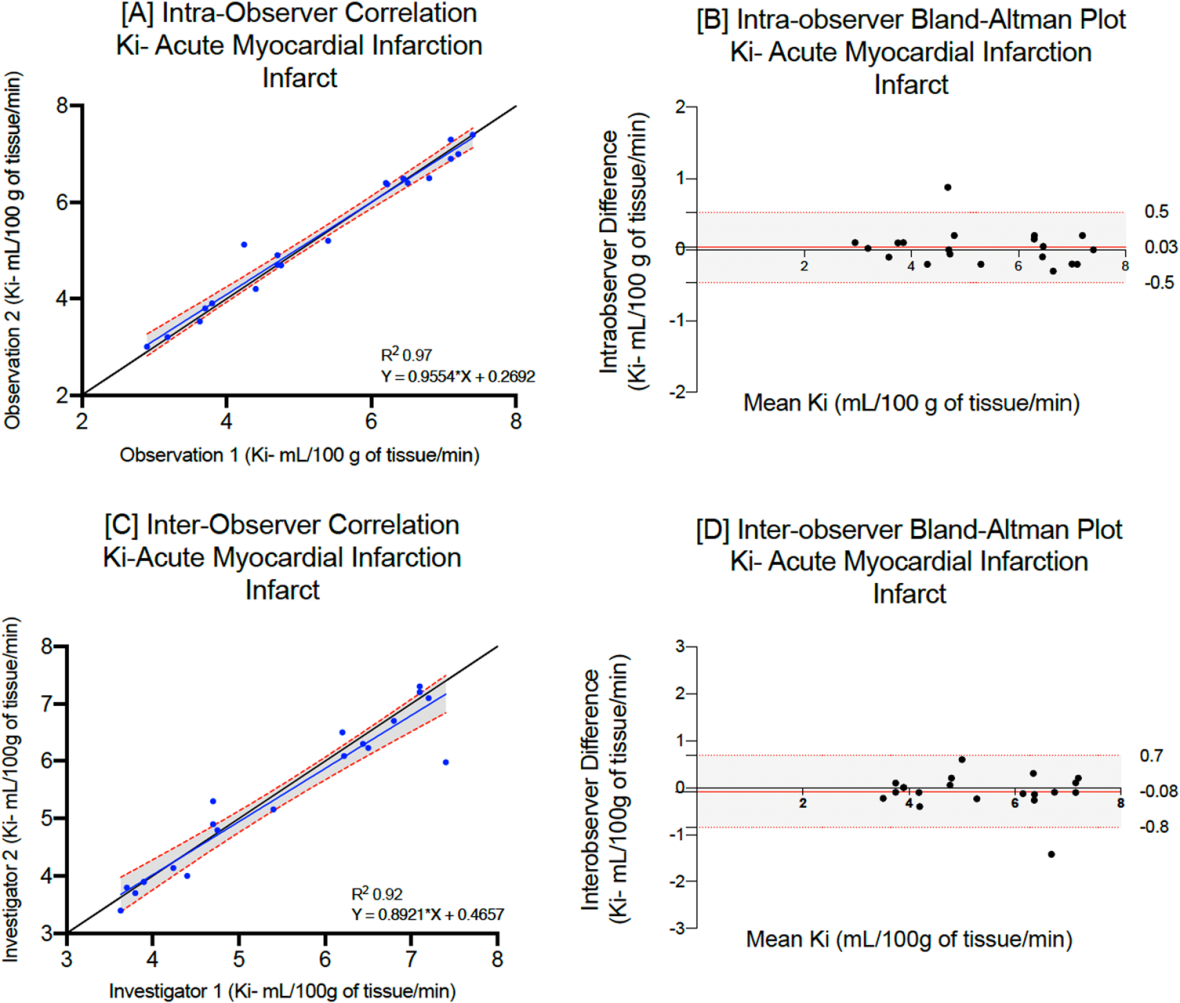
Intraobserver and interobserver repeatability of myocardial manganese uptake in acute myocardial infarction. Intra-observer linear regression analysis (**A**) and Bland–Altman plots for myocardial manganese uptake (**B**) in patients with acute myocardial infarction. Inter-observer linear regression analysis (**C**) and Bland–Altman plots for myocardial manganese uptake (**D**) in patients with acute myocardial infarction.

**Figure 4:**
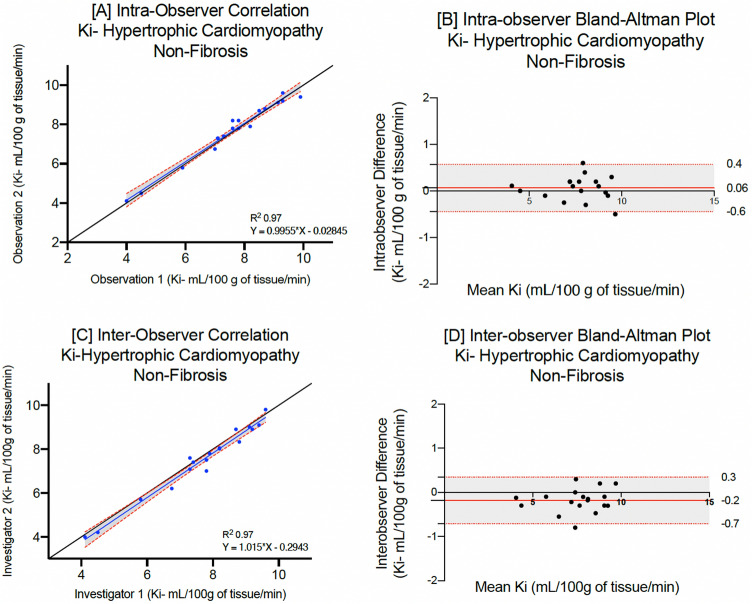
Intraobserver and interobserver repeatability of myocardial manganese uptake in hypertrophic cardiomyopathy. Intra-observer linear regression analysis (**A**) and Bland–Altman plots for myocardial manganese uptake (**B**) in patients with hypertrophic cardiomyopathy. Inter-observer linear regression analysis (**C**) and Bland–Altman plots for myocardial manganese uptake (**D**) in patients with hypertrophic cardiomyopathy.

**Figure 5:**
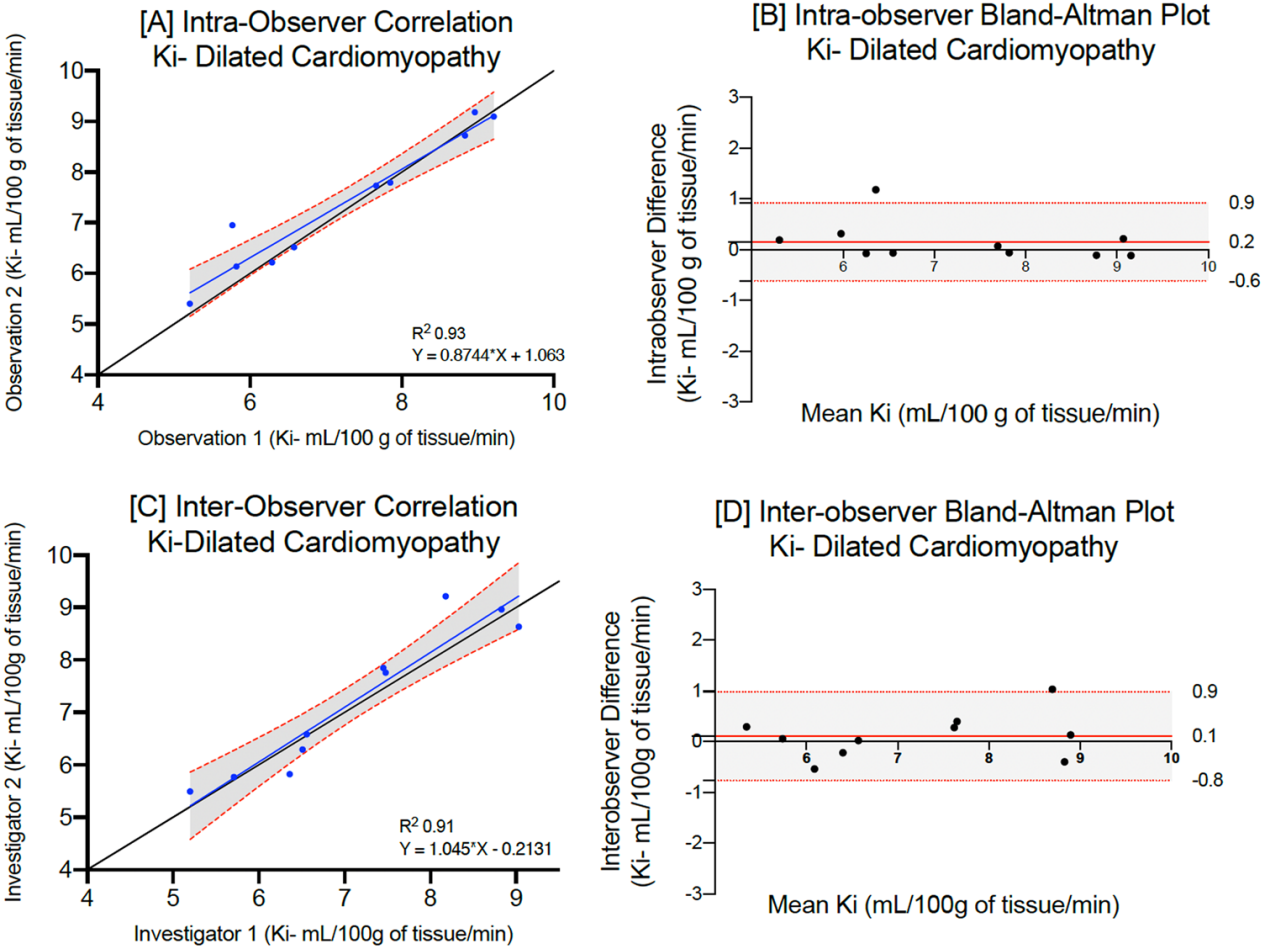
Intraobserver and interobserver repeatability of myocardial manganese uptake in dilated cardiomyopathy. Intra-observer linear regression analysis (**A**) and Bland–Altman plots for myocardial manganese uptake (**B**) in patients with dilated cardiomyopathy. Inter-observer linear regression analysis (**C**) and Bland–Altman plots for myocardial manganese uptake (**D**) in patients with dilated cardiomyopathy.

The mean native T1 for patients with acute myocardial infarction (infarct), hypertrophic (non-fibrosis) or dilated cardiomyopathy were 1395 ± 72, 1185 ± 35 and 1208 ± 60 ms respectively. On repeated analysis by the same observer, there were no differences between mean myocardial T1 values (p = 0.87, 0.96 and 0.92 respectively, Table 2). Mean myocardial manganese uptake in patients with acute myocardial infarction (infarct) hypertrophic (non-fibrosis) and dilated cardiomyopathy was 5.3 ± 1.4, 7.6 ± 1.6 and 7.2 ± 1.5 mL/100 g of tissue/min respectively. Repeated analyses demonstrated excellent correlation across all patient cohorts (Lin’s correlation coefficient: 0.98, 0.97 and 0.95 respectively, Table 2). Bland–Altman plots demonstrate excellent agreement (Figs. 3, 4 and 5), although patients with dilated cardiomyopathy demonstrated the widest limits of agreement for native, post-manganese T1 and myocardial manganese uptake (−15.6 to 11.4, −22.5 to 18.4 and −0.9 to 0.6 respectively, Table 2).

**Table 2:** Intra-observer repeatability for patients with myocardial infarction (infarct), hypertrophic cardiomyopathy (non-fibrosis) or dilated cardiomyopathy.

Myocardial infarction (*n* = 20)	First measurement	Second measurement	Mean difference	P value	LCC (95% CI)	Bland–Altman (95% LoA)	CoV (%)
Native T1 (ms) infarct	1395 ± 72 [1246–1501]	1391 ± 74 [1241–1485]	−3.8	0.87	0.97 (0.91–0.99)	−20.0 (−27.4, −13.1) to 12.9 (5.8, 20.1)	4.1
30-min post manganese T1 (ms) infarct	1137 ± 79 [996–1280]	1133 ± 80 [1009–1284]	−4.2	0.87	0.97 (0.91–0.99)	− 18.4 (−24.8, −12.7) to 8.9 (2.86, 14.82)	6.9
Manganese uptake (Ki, mL/100 g of tissue/min) infarct	5.3 ± 1.4 [2.0–7.3]	5.4 ± 1.4 [3.0–7.4]	+ 0.06	0.95	0.98 (0.94–0.99)	− 0.4 (−0.8, −0.3) to 0.6 (0.3, 0.7)	8.1

Hypertrophic cardiomyopathy (*n* = 18)							
Native T1 (ms) non fibrosis	1185 ± 35 [1104–1237]	1188 ± 33 [1112–1240]	+ 2.9	0.96	0.98 (0.93–0.99)	− 15.1 (−20.7, −9.5) to 9.2 (3.6, 14.7)	2.9
30-min post manganese T1 (ms) non fibrosis	922 ± 44 [838–994]	919 ± 40 [848–980]	−3.0	0.80	0.95 (0.81–0.98)	− 11.2 (−17.4, −4.8) to 15.1 (8.83, 21.4)	4.1
Manganese uptake (Ki, mL/100 g of tissue/min)	7.6 ± 1.6 [4.0–9.9]	7.7 ± 0.7 [7.4–9.4]	+ 0.07	0.96	0.97 (0.93–0.99)	− 0.6 (−0.8, −0.1) to 0.4 (0.2, 0.7)	7.4

Dilated cardiomyopathy (*n* = 10)							
Native T1 (ms)	1208 ± 6 [1164–1252]	1206 ± 61 [1162–1250]	−2.7	0.92	0.99 (0.97–1.0)	−15.6 (−25.6, −6.1) to 11.4 (1.78, 20.9)	5.9
30-min post manganese T1 (ms)	981 ± 58 [923–1039]	985 ± 61 [924–1046]	+ 5.1	0.85	0.97 (0.83–0.97)	−22.5 (−25.5, −13.2) 18.4 (1.1, 24.5)	6.3
Manganese uptake (Ki, mL/100 g of tissue/min) non fibrosis	7.2 ± 1.5 [6.2–8.3]	7.4 ± 1.3 [6.4–8.3]	+ 0.2	0.81	0.95 (0.84–0.98)	− 0.9 (−1.5, −0.4) to 0.6 (0.04, 1.16)	12.5

Mean ± standard deviation [95% confidence interval].

*LCC* Lin’s concordance correlation, *LoA* limits of agreement, *CoV* coefficient of variation.

There was no difference in mean native T1 and myocardial manganese uptake in patients with acute myocardial infarction (peri-infarct and remote) and hypertrophic cardiomyopathy (fibrosis) on repeat analysis. Similarly, there was excellent correlation and Bland–Altman plots demonstrate narrow limits of agreement (Supplementary Table S1). Peri-infarct regions in patients with acute myocardial infarction and fibrotic regions in patient with hypertrophic cardiomyopathy demonstrated wider limits of agreement compared to infarct region and non-fibrotic regions in the same cohorts (Table 2).

#### Inter-observer repeatability

Similar levels of interobserver repeatability were also seen for patients with acute myocardial infarction, hypertrophic or dilated cardiomyopathy (Table 3, Figs. 3, 4 and 5). Across all cohorts, coefficient of variation was higher for myocardial manganese uptake compared to native T1. Similarly, there was excellent correlation with narrow limits of agreement in peri-infarct and remote myocardium in patients with acute myocardial infarction and fibrotic myocardium in patients with hypertrophic cardiomyopathy (Supplementary Table S2).

**Table 3:** Inter-observer repeatability for patients with myocardial infarction (infarct), hypertrophic cardiomyopathy (non-fibrosis) or dilated cardiomyopathy.

Myocardial infarction (*n* = 20)	First observer	Second observer	Mean Difference	P value	LCC (95% CI)	Bland–Altman (95% LoA)	CoV (%)
Native T1 (ms) infarct	1395 ± 72 [1246–1501]	1390 ± 74 [1222–1484]	−4.4	0.83	0.98 (0.70–0.97)	−21.4 (−32.2, −10.5) to 28.9 (18.1, 39.80)	5.2
30-min post manganese T1 (ms) infarct	1137 ± 79 [996–1280]	1141 ± 74 [1001–1271]	+ 3.3	0.88	0.99 (0.97–0.99)	−23.5 (−32.5, −14.7) to 17.6 (8.7, 26.5)	6.9
Manganese uptake (Ki, mL/100 g of tissue/min) infarct	5.3 ± 1.4 [2.0–7.3]	5.2 ± 1.3 [2.9–7.3]	−0.08	0.79	0.98 (0.96–0.99)	− 0.7 (−1.03, −0.4) to 0.9 (0.5, 1.2)	9.9

Hypertrophic cardiomyopathy (*n* = 18)							
Native T1 (ms) non fibrosis	1185 ± 35 [1104–1237]	1189 ± 33 [110–1206]	+ 3.9	0.88	0.97 (0.84–0.98)	−26.8 (−37.6, −16.2) to 19.1 (8.5, 27.7)	3.1
30-min post manganese T1 (ms) non fibrosis	922 ± 44 [838–994]	917 ± 45 [844–975]	−4.8	0.82	0.97 (0.94–0.99)	− 11.6 (−19.8, −3.4) to 22.7 (914.5, 31.0)	4.3
Manganese uptake (Ki, mL/100 g of tissue/min) non fibrosis	7.6 ± 1.6 [4.0–9.9]	7.4 ± 1.6 [3.09–9.8]	−0.2	0.89	0.98 (0.96–0.99)	− 0.3 (−0.6, −1.0) to 0.7 (0.5, 0.9)	8.2

Dilated cardiomyopathy (*n* = 10)							
Native T1 (ms)	1208 ± 60 [1164–1252]	1206 ± 68 [1157–1256]	−2.2	0.94	0.98 (0.94–0.99)	−17.7 (−31.2, −4.1) to 20.3 (6.7, 33.20)	5.8
30-min post manganese T1 (ms)	981 ± 58 [923–1039]	977 ± 57 [920–1034]	−4.6	0.88	0.98 (0.69–0.92)	− 20.6 (−32.3, −8.9) to 12.4 (90.65, 24.1)	6.5
Manganese uptake (Ki, mL/100 g of tissue/min)	7.2 ± 1.5 [6.2–8.3]	7.1 ± 1.3 [6.2–8.0]	−0.1	0.90	0.95 (0.79–0.99)	−0.8 (−1.1, −0.2) to 0.9 (0.2, 1.1)	13.2

Mean ± standard deviation [95% confidence interval].

*LCC* Lin’s concordance correlation, *LoA* limits of agreement, *CoV* coefficient of variation.

## Discussion

Cardiac manganese-enhanced magnetic resonance imaging holds major promise in the assessment of myocardial calcium handling. In this study of cardiac manganese-enhanced magnetic resonance imaging, we demonstrate for the first time that myocardial T1 mapping and kinetic modelling of manganese uptake is repeatable and reproducible in both healthy and diseased myocardium. We found excellent intra-observer and inter-observer repeatability as well as scan-rescan reproducibility for measures of manganese uptake in the myocardium. This suggests that this technique is sufficiently robust for application in clinical care.

As expected, septal native T1 values for healthy volunteers and patient cohorts demonstrated less variability between intra and inter observer measurements compared to post-manganese T1 values. Previous studies have described greater variation in post-contrast T1 values with gadolinium and therefore similar effects with manganese are not unexpected^26–29^. Contrast-enhanced T1 mapping is a function of contrast agent dispersion and volume of distribution which may differ across individuals. This can explain why post-manganese T1 values had greater variability compared to native T1, although these differences were small.

Coefficients of variation for myocardial manganese uptake in healthy volunteers and patient cohorts for both intra- and inter-observer repeatability and scan-rescan reproducibility were higher than for native and post-manganese T1. Kinetic modelling of myocardial manganese uptake is dependent on variables such as native T1, post-manganese T1 and blood pool signal. As such, variability in any of those factors will result in greater variability in myocardial manganese uptake. Furthermore, heterogeneities in cardiac perfusion, contrast agent kinetics and dispersion will also lead to greater bias and variability. Despite this, we observed very strong correlation with little variation in intra and inter-observer and inter-scan measurements of myocardial manganese uptake in healthy volunteers and patient cohorts.

Similar to previous cardiac magnetic resonance reproducibility studies^23,29–32^, we demonstrate that intra-observer agreements for native T1 and myocardial manganese uptake were stronger than inter-observer and scan-rescan agreements for healthy volunteers and patient cohorts. However, the main differences seem to be the 95% confidence intervals rather than the mean. Interestingly, intra-observer repeatability demonstrated broader limits of agreement in patients with dilated cardiomyopathy. This likely reflects the difficulty in defining a region of interest in the mid-septal wall in patients with dilated cardiomyopathy due to myocardial thinning. Furthermore, due to the diffuse nature of fibrosis seen in dilated cardiomyopathy, larger studies of manganese-enhanced magnetic resonance imaging would be beneficial. Despite this, there was very strong intra-observer and inter-observer correlation in T1 mapping and kinetic modelling in patients with dilated cardiomyopathy.

Compared to the other study populations and myocardial regions, peri-infarct regions in patients with acute myocardial infarction and fibrotic regions in patients with hypertrophic cardiomyopathy demonstrated broader limits of agreement and higher coefficients of variation for intra and interobserver repeatability. This likely reflects the subtle variations in discriminating peri-infarct and fibrotic regions as well as the manual delineation of endocardial and epicardial borders. Despite this, we continued to observe strong intra-observer and inter-observer correlations for such regions.

Manganese-enhanced magnetic resonance imaging has shown promise as a surrogate marker of myocardial calcium uptake in patients with ischemic and non-ischemic cardiomyopathies^16,17^ demonstrating its potential for clinical application. As such, it is important to validate this technique before use in clinical practice. Current studies are underway assessing patients at risk of developing heart failure (NCT04591639). Similar to traditional imaging with gadolinium, there is variation in measurements between vendors and different T1 mapping techniques, and further work is required to ensure consistency across different platforms and scanners.

Our study has some limitations. First, we were not able to perform scan-rescan measurements on patients with hypertrophic and dilated cardiomyopathy. It would be important to confirm that this technique has similar reproducibility in diseased states if it is to be used for serial scanning to assess disease progression or treatment interventions. Second, healthy volunteers were scanned using ShMOLLI T1 and patient cohorts underwent MOLLI T1 mapping. Our aim was to assess the reproducibility of manganese-enhanced magnetic resonance imaging in healthy and pathological myocardium. As such, we are not comparing patients with healthy volunteers. Furthermore, we have demonstrated that manganese-enhanced magnetic resonance imaging has strong intra- and inter- observer repeatability using either MOLLI or ShMOLLI T1 mapping. However, it is essential for future studies to assess this further. Third, performing a statistical test for defining cut-off for normal versus abnormal myocardial manganese uptake (Ki) was not in the scope of our study. This should be the focus of future work to define a normal reference range for these tracer kinetic parameters. Finally, manganese dipyridoxyl diphosphate is currently not readily or widely available for clinical use although we anticipate that this is likely to change in the near future.

In conclusion, although larger studies are required, our data suggest that manganese-enhanced T1 mapping and kinetic modelling is a repeatable and reproducible technique in healthy myocardium. Furthermore, this technique demonstrates high repeatability in ischaemic and non-ischaemic cardiomyopathy. However, it does require further study to assess repeatability and reproducibility in different cardiac pathologies with diffuse or subtle fibrosis, such as patients with dilated cardiomyopathy, as well as defining normal reference ranges for these novel measures of myocardial calcium handling.

## Supplementary Information

Supplementary Information.

## Data Availability

The datasets used and analysed during the current study are available from the corresponding author on reasonable request.

## Abbreviations

MOLLI: Modified look-locker inversion recovery
ShMOLLI: Shortened modified look-locker inversion recovery
CoV: Coefficient of variation
LoA: Limits of agreement
LCC: Lin’s correlation coefficient
