# Supplementary material for: Repeatability and reproducibility of cardiac manganese-enhanced magnetic resonance imaging
Source: Sci Rep. 2023 Feb 27;13:3366. doi: 10.1038/s41598-023-29591-z (PMC9971197; doi:10.1038/s41598-023-29591-z)
Supplement: Supplementary file 1 — Supplementary Information. [file 41598_2023_29591_MOESM1_ESM.docx]

**Supplementary Files**

**Repeatability and Reproducibility of**

**Cardiac Manganese-Enhanced**

**Magnetic Resonance Imaging**

**Supplementary Figure 1:**


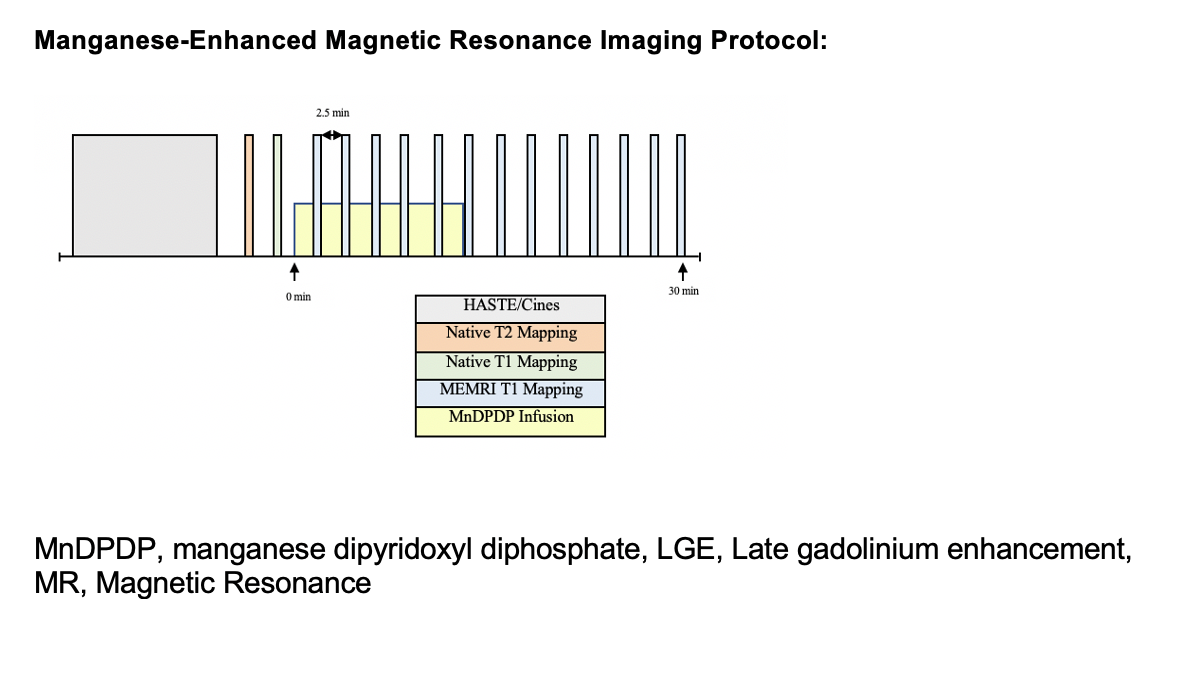


**Supplementary Figure 2**

**Patlak formulation: Patlak formulation – schematic of (A) model compartments and transfer constant K_i_ , describing passage from reversible to irreversible compartment (B) data analysis**

**
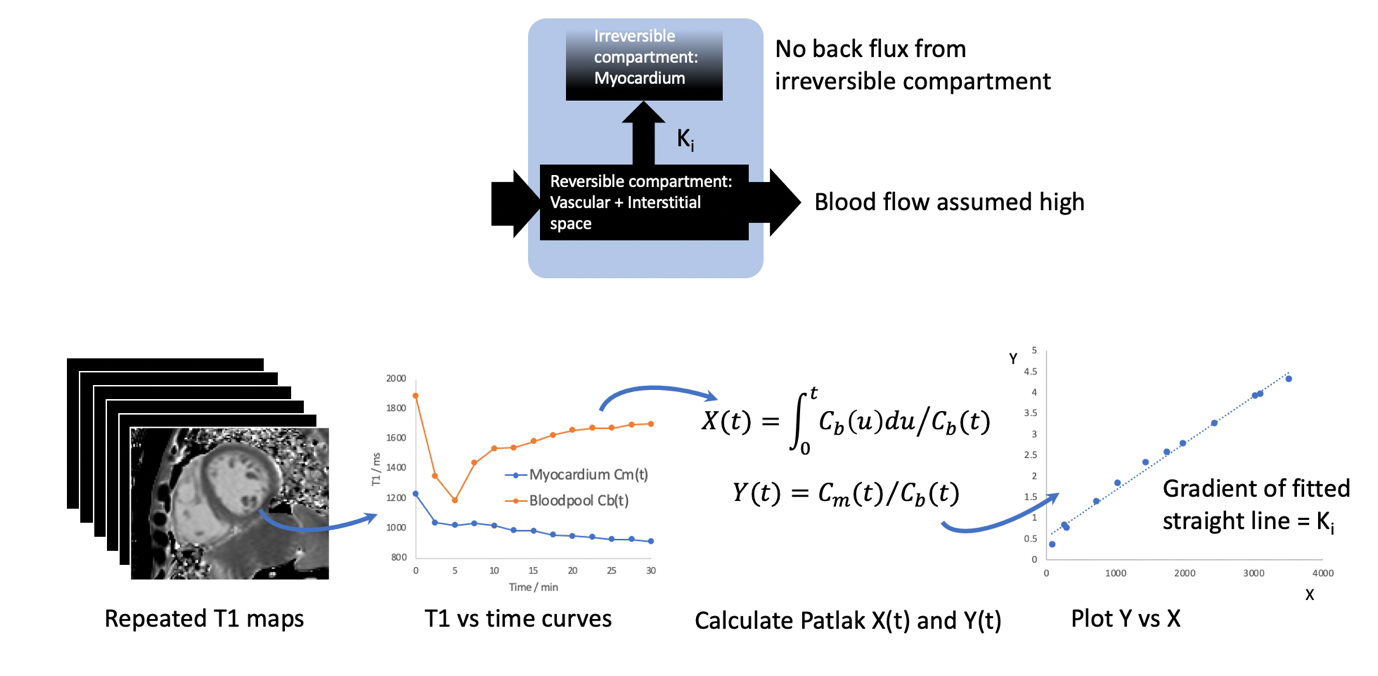
**

*k_1_* transfer into reversible compartment (transfer constant)

**Supplementary Figure 3**

1. **Mean native T1 values (m/s) and (B) mean myocardial manganese uptake (Ki-ml/100g/min) per segment in healthy volunteers (n=20). Septal and global values (C).**


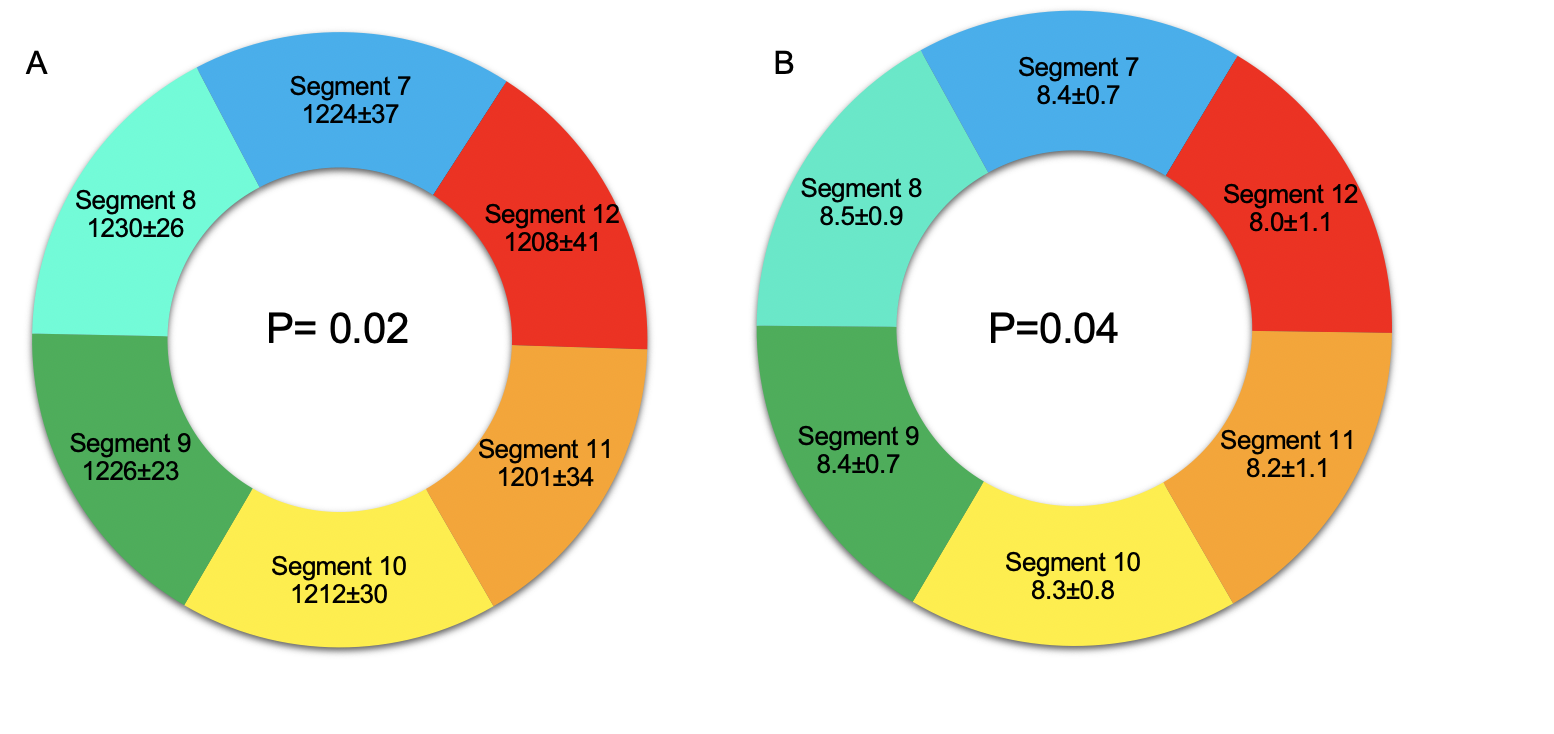


C


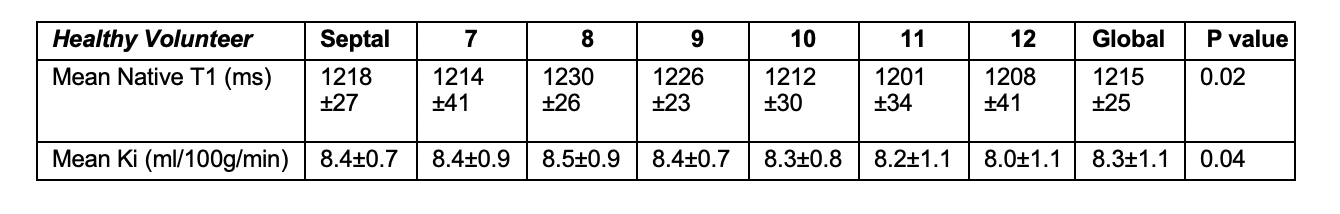


**Supplementary Table 1:** Intra-observer repeatability for patients with myocardial infarction and hypertrophic cardiomyopathy.

| ***Myocardial infarction***  ***(n=20)*** | **First measurement** | **Second measurement** | **Mean Difference** | **P value** | **LCC**  **(95% CI)** | **Bland-Altman**  **(95% LoA)** | **CoV (%)** |
| --- | --- | --- | --- | --- | --- | --- | --- |
| Native T1 (ms)  Peri-infarct | 1263±49  [1166-1361] | 1265± 50  [1169-1358] | +2.1 | 0.89 | 0.98  (0.95 – 0.99) | -20.6 (-28.7, -12.5) to 17.0 (8.9, 25.1) | 3.8 |
| Native T1 (ms)  Remote | 1147 ± 36  [1071-1218] | 1144 ± 38  [1078-1207] | -2.8 | 0.81 | 0.95  (0.88 – 0.98) | -20.4 (-30.1, -10.5) to 23.4 (15.7, 31.20 | 3.9 |
| 30 min Post Manganese T1 (ms)  Peri-infarct | 1009±20  [973-1058] | 1010±21  [971-1041] | +1.6 | 0.86 | 0.90  (079 – 0.96) | -13.1 (-27.7, -10.5) to 22.1 (13.2, 31.0) | 3.1 |
| 30 min Post Manganese T1 (ms) Remote | 886 ± 37  [816 – 944] | 889 ± 34  [812 – 947] | +2.2 | 0.83 | 0.94  (0.86-0.97) | -17.6 (27.8, 16.30 to 21.6 (11.1, 32.2) | 4.4 |
| Manganese uptake- (Ki, mL/100 g of tissue/min)- Peri infarct | 6.7 ±1.2  [4.0-8.6] | 6.5 ± 1.1  [4.1-8.6] | -0.09 | 0.79 | 0.92  (0.80-0.96) | -0.8 (-1.2, -0.40) to 1.0 (0.6, 1.4) | 17.3 |
| Manganese uptake- (Ki, mL/100 g of tissue/min)- Remote | 8.6±1.2  [6.0-11] | 8.7±1.3  [6.0-11.3] | +0.05 | 0.90 | 0.98  (0.96-0.99) | -0.5 (-0.6, -0.2) to 0.4 (0.2, 0.5) | 15.3 |
| ***Hypertrophic cardiomyopathy***  ***(n=18)*** |  |  |  |  |  |  |  |
| Native T1 (ms)  Fibrosis (n=11) | 1332 ±91  [1219 – 1477] | 1335 ± 86  [1224 – 1465] | + 3.0 | 0.93 | 0.97  (0.92-0.98) | -11.8 (22.0, -1.6) to 19.2 (9.0, 29.3) | 3.4 |
| 30 min Post Manganese T1 (ms) Fibrosis (n=11) | 1083 ± 87  [1006-1261] | 1086 ±90  [1010-1251] | +3.7 | 0.95 | 0.94  (0.79-0.98) | -17.7 (-36.7, -8.9) to 9.7 (0.7, 18.7) | 4.3 |
| Manganese uptake  (Ki, mL/100 g of tissue/min)  Fibrosis (n=11) | 5.2 ±1.5  [3.2-7.3] | 5.1 ± 1.2  [3.0 – 7.4] | -1.0 | 0.88 | 0.97  (0.92-0.98) | -0.3 (-0.5, -0. 2) to 0.1 (-0.5, 0.2) | 14.1 |

Mean± standard deviation [95% confidence interval], LCC, Lin’s concordance correlation, LoA, limits of agreement, CoV, coefficient of variation.

**Supplementary Table 2:** Inter-observer repeatability for patients with myocardial infarction and hypertrophic cardiomyopathy.

| ***Myocardial infarction***  ***(n=20)*** | **First observer** | **Second observer** | **Mean Difference** | **P value** | **LCC**  **(95% CI)** | **Bland-Altman**  **(95% LoA)** | **CoV (%)** |
| --- | --- | --- | --- | --- | --- | --- | --- |
| Native T1 (ms)  Peri-infarct | 1263±49  [1166-1361] | 1267±50  [1169-1373] | +4.1 | 0.87 | 0.97  (0.92 – 0.99) | -33.6 (-43.7, -5.1) to 28.2 (14.9, 41.3) | 4.9 |
| Native T1 (ms)  Remote | 1147 ± 36  [1071-1218] | 1144 ± 34  [1071 – 1209] | -2.6 | 0.82 | 0.89  (0.68- 0.97) | -19.4 (-29.3, 9.80 to 25.04 (15.6, 35.1) | 3.5 |
| 30 min Post Manganese T1 (ms)  Peri-infarct | 1010 ± 20  [973-1058] | 1015 ± 25  [971-1058] | +4.6 | 0.65 | 0.88  (0.60 – 0.96) | -28 (-36.5, -14.3) to 19.3 (13.5, 32.6) | 7.1 |
| 30 min Post Manganese T1 (ms) Remote | 886 ± 37  [816 – 944] | 882 ± 41  [809 – 941] | -4.9 | 0.72 | 0.86  (0.55 – 0.94) | -21.4 (-26.5, -9.5) to 31.6 (22.6, 54.3) | 6.4 |
| Manganese uptake- (Ki, mL/100 g of tissue/min)- Peri infarct | 6.7 ±1.2  [4.0-8.6] | 6.5 ± 1.1  [3.2-8.3] | - 0.09 | 0.82 | 0.94  (0.80 – 0.98) | -0.9 (-1.37, -0.5) to 1.1 (0.6, 1.6) | 17.8 |
| Manganese uptake- (Ki, mL/100 g of tissue/min)- Remote | 8.6±1.2  [6.0-11] | 8.5 ±1.2  [6.3-10.2] | -0.04 | 0.88 | 0.97  (0.90 – 0.99) | -0.5 (-0.7, -.3) to 0.6 (0.4, 0.8) | 16.7 |
| ***Hypertrophic cardiomyopathy***  ***(n=18)*** |  |  |  |  |  |  |  |
| Native T1 (ms)  Fibrosis (n=11) | 1332 ±91  [1219 – 1477] | 1337 ± 86  [1221 – 1476] | + 5.3 | 0.89 | 0.94  (0.90-0.98) | -24.4 (-43.7, -5.1) to 43.2 (14.9, 53.3) | 6.5 |
| 30 min Post Manganese T1 (ms) Fibrosis (n=11) | 1083 ± 87  [1006-1261] | 1079 ± 90  [1001-1249] | -4.9 | 0.95 | 0.94  (0.79-0.98) | -18.4 (-24.3, -9.4) to 23.2 (11.5, 31.3) | 7.3 |
| Manganese uptake  (Ki, mL/100 g of tissue/min)  Fibrosis (n=11) | 5.2 ±1.5  [3.2-7.3] | 5.3 ± 1.4  [3.2 – 7.5] | +0.2 | 0.80 | 0.97  (0.92-0.99) | -0.5 (-0.8, 0.3) to 0.2 (-0.02, 0.4) | 16.1 |

Mean± standard deviation [95% confidence interval], LCC, Lin’s concordance correlation, LoA, limits of agreement, CoV, coefficient of variation.
